# External Validation of Fatty Liver Index for Identifying Ultrasonographic Fatty Liver in a Large-Scale Cross-Sectional Study in Taiwan

**DOI:** 10.1371/journal.pone.0120443

**Published:** 2015-03-17

**Authors:** Bi-Ling Yang, Wen-Chieh Wu, Kuan-Chieh Fang, Yuan-Chen Wang, Teh-Ia Huo, Yi-Hsiang Huang, Hwai-I Yang, Chien-Wei Su, Han-Chieh Lin, Fa-Yauh Lee, Jaw-Ching Wu, Shou-Dong Lee

**Affiliations:** 1 Division of Gastroenterology, Department of Medicine, Taipei Veterans General Hospital, Taipei, Taiwan; 2 Division of Gastroenterology, Department of Medicine, Cheng Hsin General Hospital, Taipei, Taiwan; 3 Division of Gastroenterology, Department of Medicine, Ren-Ai Branch, Taipei City Hospital, Taipei, Taiwan; 4 Faculty of Medicine, School of Medicine, National Yang-Ming University, Taipei, Taiwan; 5 Healthcare Center, Taipei Veterans General Hospital, Taipei, Taiwan; 6 Department and Institute of Pharmacology, National Yang-Ming University, Taipei, Taiwan; 7 Institute of Clinical Medicine, School of Medicine, National Yang-Ming University, Taipei, Taiwan; 8 Genomics Research Center, Academia Sinica, Taipei, Taiwan; 9 Division of Translational Research, Department of Medical Research, Taipei Veterans General Hospital, Taipei, Taiwan; Institute of Medical Research A Lanari-IDIM, University of Buenos Aires-National Council of Scientific and Technological Research (CONICET), ARGENTINA

## Abstract

**Background and Aims:**

The fatty liver index (FLI) is an algorithm involving the waist circumference, body mass index, and serum levels of triglyceride and gamma-glutamyl transferase to identify fatty liver. Although some studies have attempted to validate the FLI, few studies have been conducted for external validation among Asians. We attempted to validate FLI to predict ultrasonographic fatty liver in Taiwanese subjects.

**Methods:**

We enrolled consecutive subjects who received health check-up services at the Taipei Veterans General Hospital from 2002 to 2009. Ultrasonography was applied to diagnose fatty liver. The ability of the FLI to detect ultrasonographic fatty liver was assessed by analyzing the area under the receiver operating characteristic (AUROC) curve.

**Results:**

Among the 29,797 subjects enrolled in this study, fatty liver was diagnosed in 44.5% of the population. Subjects with ultrasonographic fatty liver had a significantly higher FLI than those without fatty liver by multivariate analysis (odds ratio 1.045; 95% confidence interval, CI 1.044–1.047, p< 0.001). Moreover, FLI had the best discriminative ability to identify patients with ultrasonographic fatty liver (AUROC: 0.827, 95% confidence interval, 0.822–0.831). An FLI < 25 (negative likelihood ratio (LR−) 0.32) for males and <10 (LR− 0.26) for females rule out ultrasonographic fatty liver. Moreover, an FLI ≥ 35 (positive likelihood ratio (LR+) 3.12) for males and ≥ 20 (LR+ 4.43) for females rule in ultrasonographic fatty liver.

**Conclusions:**

FLI could accurately identify ultrasonographic fatty liver in a large-scale population in Taiwan but with lower cut-off value than the Western population. Meanwhile the cut-off value was lower in females than in males.

## Introduction

Fatty liver disease has become an emerging public health concern because its prevalence and incidence rates have rapidly increased in recent decades [1,2]. With different study populations and diagnostic tools, the prevalence rate of fatty liver disease has been reported to be 10–35% in the United States. Fatty liver is correlated with metabolic factors such as central obesity, insulin resistance, arterial hypertension, and hypertriglyceridemia [3,4]. Due to the Westernization of diet and lifestyle and the aging population, the prevalence rate of fatty liver is also increasing in Asian countries. Large population-based surveys in China, Japan, Korea, and Taiwan indicate that the prevalence of fatty liver disease now stands at 12% to 51% in population subgroups, depending on age, gender, ethnicity, and social-economic status [2,5–7].

Moreover, fatty liver disease is now the leading cause of abnormal liver biochemistry tests in the primary care setting worldwide [8]. The clinic-pathological spectrum of fatty liver disease ranges from simple steatosis to steatohepatitis, which may progress to liver cirrhosis and hepatocellular carcinoma (HCC) [9]. The prevalence of fatty liver-related cirrhosis has markedly increased in recent years as the underlying liver disease among patients undergoing transplants for HCC in the United States [10,11]. Welzel et al. further demonstrated that diabetes and/or obesity had the largest population-attributable fractions of HCC, with a value of 36.6% [12]. This rate is significantly higher than that of viral hepatitis, suggesting a dominant role of fatty liver and metabolic disorders for hepatic carcinogenesis.

Most subjects with fatty liver do not have specific symptoms, especially at the early stage, which limits prevention and early detection of fatty liver disease [13]. Liver biopsy is regarded as the gold standard for quantification of liver steatosis in fatty liver disease [14]. However, it is not routinely performed because it is an invasive procedure with a significant degree of sampling error. Hence, the diagnosis of fatty liver in the population studies is usually made by ultrasonography [9]. More sensitive techniques, including magnetic resonance imaging and spectroscopy, are hindered by expense and unfeasibility for large populations [15].

Bedogni et al. established a formula to calculate the fatty liver index (FLI) based on triglycerides (TG), body mass index (BMI), gamma-glutamyltrasnferase (GGT), and waist circumference (WC) to predict ultrasonogrphic fatty liver in an Italian cohort [16]. This simple and non-invasive algorithm has excellent discriminative ability to detect ultrasonogrphic fatty liver disease. Nevertheless, few studies have been conducted for the external validation of FLI in Asians thus far [17]. We attempted to validate FLI for the prediction of ultrasonogrphic fatty liver in Taiwanese subjects and compared FLI with lipid accumulation products (LAP) which is recently considered as a good marker of liver steatosis [18]. We also attempted to determine the optimal cut-off levels of FLI in detecting ultrasonogrphic fatty liver and stratified them by gender.

## Materials and Methods

### Study population

There were 34,346 consecutive examinees receiving health check-up services at the Taipei Veterans General Hospital from 2002 to 2009. Subjects who had chronic hepatitis C virus (HCV) infection (n = 819), chronic hepatitis B virus (HBV) infection (n = 3,642), and dual HBV/HCV infections (n = 88) were excluded. The remaining 29,797 subjects were included in the final analysis.

All of the subjects underwent a complete clinical evaluation, laboratory examination and abdominal ultrasonography. BMI was calculated by the division of the body weight in kilograms by the square of body height in meters. In this study, all ultrasonography were performed by five senior doctors with more than 10 years of experience and fatty liver was diagnosed according to the criteria from the American Gastroenterology Association including (1) a diffuse hyperechoic echotexture, (2) increased liver echotexture compared with the kidneys, (3) vascular blurring, and (4) deep attenuation [19]. One recent meta-analysis conducted by Hernaez and colleagues showed that ultrasonography had reliable and accurate detection of fatty liver [20]. Moreover, Dasarathy demonstrated that the increased hepato-renal contrast and bright liver were able to identify the presence of ≧20% area of involvement with fat with a sensitivity of 96.4% and a specificity of 97.8% [21]. The study followed the standards of the Declaration of Helsinki and has been approved by the Institutional Review Board (IRB) of the Taipei Veterans General Hospital. As the dataset used in this study is consisted of de-identified data from a retrospective cohort, the written informed consents from the subjects who receiving physical check-up services were waived by the approval of the IRB.

### Biochemical and serologic markers

Venous blood samples were collected after an overnight fast. Radio-immunoassay (Abbott Laboratories, North Chicago, IL, USA) was used to test serum HBV surface antigen (HBsAg), and second-generation enzyme immunoassay (Abbott Laboratories) was used to test antibody to HCV (anti-HCV). The Roche/Hitachi Modular Analytics System (Roche Diagnostics GmbH, Mannheim, Germany) was utilized to measure serum biochemical markers. FLI was calculated using the following formula: FLI = (e 0.953*loge (TG) + 0.139*BMI + 0.718*loge (GGT) + 0.053*WC—15.745) / (1 + e 0.953*loge (TG) + 0.139*BMI + 0.718*loge (GGT) + 0.053*WC—15.745) * 100 [16]. LAP was calculated with formula: LAP = (waist circumference (cm) – 58) × triglycerides (mmol/l) [18].

### Statistical analysis

Pearson’s chi-squared analysis and Student t-test analysis were performed to compare categorical and continuous variables between subjects with and without ultrasonogrphic fatty liver. Variables with statistical significance (P < 0.05) or proximate to it (P < 0.1) in univariate analysis were further included in multivariate analysis using a logistic regression model with the forward stepwise selection procedure. The performance of serum markers in diagnosing ultrasonogrphic fatty liver was examined using the area under the receiver operator characteristic (AUROC) curves. The AUROC was expressed as plots of the test sensitivity vs. 1—specificity. The sensitivity, specificity, positive likelihood ratio (LR+), negative likelihood ratio (LR−), positive predictive value (PPV), and negative predictive value (NPV) were also assessed. A P value of <0.05 was considered to be statistically significant. All statistical analyses were performed using SPSS 17.0 for Windows (SPSS Inc., Chicago, IL, USA).

## Results

### Demographic characteristics of study subjects

Among the 29,797 subjects that were enrolled in this study, the mean age was 52.2 years, and 54.0% were male. Fatty liver was diagnosed by ultrasonography in 44.5% of the whole population. As summarized in **Table 1**, compared to subjects without ultrasonogrphic fatty liver, those with fatty liver tended to be older and male, and they had higher BMI, larger WC, higher systolic and diastolic blood pressure (BP), higher levels of fasting glucose, cholesterol, low density lipoprotein (LDL), TG, alanine aminotransferase (ALT), aspartate aminotransferase (AST), and GGT, higher platelet counts, and higher FLI, but lower high-density lipoprotein (HDL) levels according to univariate analysis.

**Table 1:** Comparison of demographic characteristics between subjects with and without ultrasonogrphic fatty liver.

	All (n = 29797)	Without fatty liver (n = 16542)	With fatty liver (n = 13255)	P
**Age, years**	52.2±13.3	50.9±14.1	53.9±11.9	<0.001
**Sex, (M/F) (%)**	16098/13699 (54.0%/46.0%)	7388/9154 (44.7%/55.3%)	8710/4545 (65.7%/34.3%)	<0.001
**BMI, kg/m** ^**2**^	23.82±3.58	22.32±2.90	25.68±3.47	<0.001
**WC, cm**	83.8±1.3	79.5±9.0	89.1±9.3	<0.001
**SBP, mmHg**	124.3±18.6	121.1±18.5	128.2±18.0	<0.001
**DBP, mmHg**	77.5±14.3	75.3±11.6	80.4±16.6	<0.001
**Fasting Glucose, mg/dL**	95.5±24.8	90.7±19.1	101.6±29.3	<0.001
**Cholesterol, mg/dL**	199.2±37.0	194.0±36.0	205.0±37.5	<0.001
**LDL, mg/dL**	125.3±32.9	120.5±31.7	131.4±33.2	<0.001
**HDL, mg/dL**	53.7±15.0	58.2±15.5	48.1±12.3	<0.001
**TG, mg/dL**	130.4±88.1	101.0±57.2	167.1±104.6	<0.001
**ALT, U/L**	27.0±22.2	21.2±17.2	34.3±25.3	<0.001
**AST, U/L**	23.1±13.2	21.1±11.4	25.5±14.8	<0.001
**GGT, IU/L**	24.8±36.8	20.0±33.9	30.8±39.4	<0.001
**Platelet, 1000/mm** ^**3**^	249.83±60.33	247.62±60.90	252.56±59.50	<0.001
**FLI**	27.23±19.50	15.61±16.27	41.75±24.58	<0.001
**LAP**	32.29±12.55	26.58±10.24	30.42±11.47	<0.001

Continuous variables are expressed as mean ± standard deviation

Abbreviations: M, male; F, female; BMI, body mass index; WC, waist circumference; SBP, systolic blood pressure; DBP, diastolic blood pressure; LDL, low-density lipoprotein; HDL, high-density lipoprotein; TG, triglyceride; ALT, alanine aminotransferase; AST, aspartate aminotransferase; GGT, gamma-glutamyl transferase; FLI, fatty liver index; LAP, lipid accumulation products

**Fig 1:**
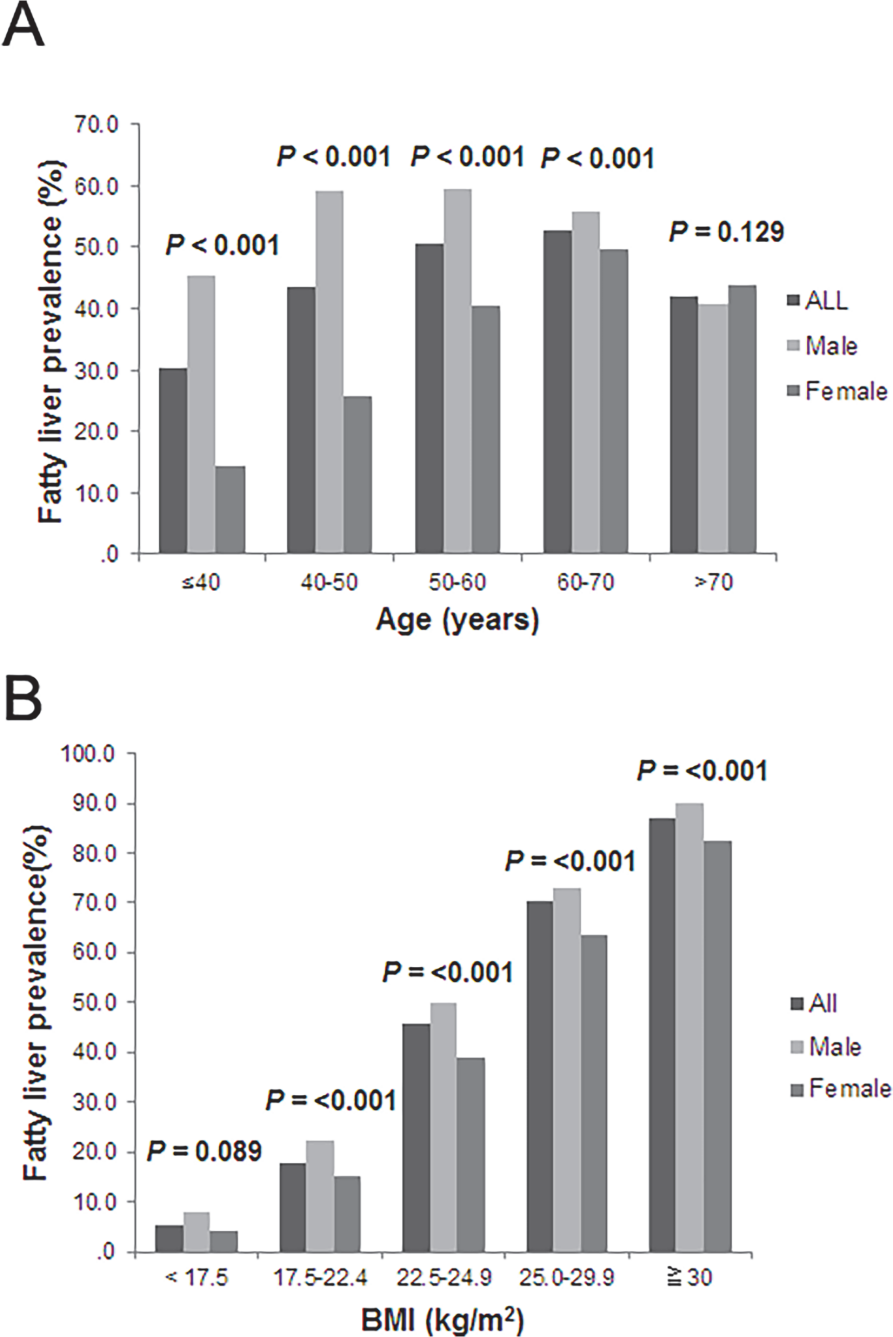
Comparison of the prevalence rate of ultrasonogrphic fatty liver between male and female subjects. **(A)** Comparison of ultrasonogrphic fatty liver prevalence rate between male and female subjects stratified by age. **(B)** Comparison of ultrasonogrphic fatty liver prevalence rate between male and female subjects stratified by body mass index.

Compared to females, male subjects were older, and they had higher BMI, larger WC, higher systolic and diastolic BP, higher levels of fasting glucose, LDL, TG, ALT, AST, and GGT, but lower platelet counts, serum cholesterol, and HDL levels **(S1 Table).** Moreover, male subjects had a significantly higher rate of fatty liver diagnosed by ultrasonography (54.1% vs. 33.2%, *p*< 0.001) and higher FLI (mean ± standard deviation, 35.35 ± 24.68 vs. 17.70 ± 19.70, *p*< 0.001) than female subjects. When stratified by age and BMI, the prevalence rates of ultrasonogrphic fatty liver were significantly higher in male subjects than in females in most of the age populations except those who were older than 70 years or those with BMI < 17.5 kg/m^2^ **(Fig. 1 and S2 Table).**

### Factors associated with ultrasonogrphic fatty liver in different populations by multivariate analysis

As FLI is calculated by the combination of BMI, WC, serum TG, and GGT levels, we applied two models in multivariate analysis to minimize the potential confounding effects of these parameters. In model I, FLI was enrolled, but its 4 components were not entered into multivariate analysis. In model II, we selected BMI, WC, TG, and GGT for the multivariate analysis, but not FLI.

As shown in **Table 2**, the multivariate analysis in model I demonstrated that older age, higher fasting glucose, LDL, and ALT levels, higher platelet counts, higher FLI, and low HDL levels were the independent risk factors correlated with ultrasonogrphic fatty liver in the whole population. In model II, higher BMI, WC, higher fasting glucose, LDL, TG, GGT levels, and lower HDL levels were associated with ultrasonogrphic fatty liver in multivariate analysis **(S3 Table).** The results were similar when the analyses were stratified by gender in both model I and model II. This indicated that FLI and its components were all critical factors for determining ultrasonogrphic fatty liver, irrespective of gender.

**Table 2:** Factors associated with ultrasonogrphic fatty liver in different populations by multivariate analysis in model I.

	Odds Ratio	95% Confidence level	*P* value
**All subjects**			
Age, years	1.007	1.004–1.009	<0.0001
Fasting glucose, mg/dL	1.009	1.007–1.010	<0.0001
LDL, mg/dL	1.006	1.005–1.007	<0.0001
ALT, U/L	1.017	1.015–1.020	<0.0001
Platelet, 1000/mm^3^	1.002	1.002–1.003	<0.0001
HDL, mg/dL	0.980	0.978–0.983	<0.0001
FLI	1.045	1.044–1.047	<0.0001
**Females**			
Age, years	1.013	1.009–1.018	<0.0001
Fasting glucose, mg/dL	1.011	1.008–1.013	<0.0001
LDL, mg/dL	1.006	1.005–1.008	<0.0001
ALT, U/L	1.016	1.013–1.020	<0.0001
Platelet, 1000/mm^3^	1.003	1.002–1.004	<0.0001
HDL, mg/dL	0.978	0.975–0.981	<0.0001
FLI	1.053	1.050–1.056	<0.0001
**Males**			
Fasting glucose, mg/dL	1.007	1.009–1.009	<0.0001
LDL, mg/dL	1.005	1.004–1.006	<0.0001
ALT, U/L	1.018	1.015–1.021	<0.0001
Platelet, 1000/mm^3^	1.002	1.001–1.002	<0.0001
HDL, mg/dL	0.982	0.979–0.985	<0.0001
FLI	1.043	1.041–1.045	<0.0001

Abbreviations: SBP, systolic blood pressure; LDL, low-density lipoprotein; HDL, high-density lipoprotein; TG, triglyceride; ALT, alanine aminotransferase; FLI, fatty liver index

### Validation of FLI for identifying ultrasonogrphic fatty liver and selection of its optimal cut-off value

Subsequently, we compared the discriminative ability to identify ultrasonogrphic fatty liver among FLI and other clinical non-invasive markers by comparing their AUROCs. As depicted in **Table 3**, FLI had the highest AUROC with a value of 0.827 (95% confidence interval, CI 0.822–0.831) in comparison to other single markers, such as BMI, LAP, GGT, TG, WC, ALT, fasting glucose, cholesterol, LDL and HDL. When analyses were stratified by gender, the AUROCs of FLI in predicting ultrasonogrphic fatty liver were 0.827 (95% CI 0.820–0.834) and 0.800 (95% CI 0.793–0.806) for females and males, respectively. They were both higher than those of the other markers, suggesting FLI had the best discriminative ability to predict ultrasonogrphic fatty liver compared to other non-invasive markers in both genders.

**Table 3:** Comparison of AUROCs among non-invasive markers for predicting ultrasonogrphic fatty liver.

	AUROC	95% CI	Standard error	*P* value
**All subjects**				
FLI	0.827	0.822–0.831	0.002	<0.0001
LAP	0.806	0.801–0.811	0.003	<0.0001
BMI	0.787	0.782–0.791	0.003	<0.0001
WC	0.777	0.771–0.782	0.003	<0.0001
ALT	0.741	0.735–0.746	0.003	<0.0001
GGT	0.715	0.710–0.720	0.003	<0.0001
Fasting glucose	0.663	0.658–0.669	0.003	<0.0001
Cholesterol	0.582	0.575–0.588	0.003	<0.0001
TG	0.755	0.750–0.761	0.003	<0.0001
HDL	0.702	0.696–0.708	0.003	<0.0001
LDL	0.597	0.591–0.604	0.003	<0.0001
**Female**				
FLI	0.827	0.820–0.834	0.004	<0.0001
LAP	0.794	0.786–0.802	0.004	<0.0001
BMI	0.786	0.778–0.794	0.004	<0.0001
WC	0.762	0.754–0.770	0.004	<0.0001
ALT	0.717	0.708–0.726	0.005	<0.0001
GGT	0.708	0.699–0.717	0.005	<0.0001
Fasting glucose	0.708	0.698–0.717	0.005	<0.0001
Cholesterol	0.599	0.589–0.609	0.005	<0.0001
TG	0.759	0.750–0.767	0.004	<0.0001
HDL	0.690	0.681–0.700	0.005	<0.0001
LDL	0.622	0.613–0.632	0.005	<0.0001
**Male**				
FLI	0.800	0.793–0.806	0.003	<0.0001
LAP	0.785	0.778–0.792	0.004	<0.0001
BMI	0.760	0.752–0.767	0.004	<0.0001
WC	0.754	0.746–0.761	0.004	<0.0001
ALT	0.725	0.717–0.732	0.004	<0.0001
GGT	0.675	0.667–0.683	0.004	<0.0001
Fasting glucose	0.616	0.607–0.625	0.004	<0.0001
TG	0.720	0.712–0.728	0.004	<0.0001
Cholesterol	0.581	0.572–0.590	0.004	<0.0001
HDL	0.657	0.649–0.664	0.004	<0.0001
LDL	0.572	0.563–0.581	0.005	<0.0001

Abbreviations: AUROC: area under the receiver operating characteristic; CI: confidence interval; FLI: fatty liver index; LAP, lipid accumulation products; BMI, body mass index; GGT, gamma-glutamyl transferase; TG: triglyceride; WC, waist circumference; ALT: alanine aminotransferase; LDL: low-density lipoprotein

As shown in **Table 4**, by using Bedogni’s method [16], the two cut-off points for the males are FLI <25 (Sensitivity 78.5%, Specificity 67.0%, LR+ 2.38, LR− 0.32) for exclusion and FLI ≥35 (Sensitivity 63.0%, Specificity 79.8%, LR+ 3.12. LR−: 0.46) for inclusion of ultrasonogrphic fatty liver. For the females, the two cut-off values are FLI <10 (Sensitivity 81.9%, Specificity 68.4%, LR+ 2.59, LR− 0.26) to rule out ultrasonogrphic fatty liver and FLI ≥20 (Sensitivity 62.0%, Specificity 86.0%, LR+ 4.43, LR− 0.44) to rule in ultrasonogrphic fatty liver, respectively.

**Table 4:** Selecting the optimal cut-off value of FLI in identifying ultrasonogrphic fatty liver stratified by gender.

Cut-off	Sensitivity (%)	Specificity (%)	LR+	LR−	DOR	PPV (%)	NPV (%)
**Female**							
05	91.79	43.84	1.63	0.19	8.57	44.8	91.5
**10**	**81.9**	**68.4**	**2.59**	**0.26**	**9.96**	**56.3**	**88.4**
15	71.8	79.5	3.50	0.35	10	63.5	87.6
**20**	**62.0**	**86.0**	**4.43**	**0.44**	**10.06**	**68.7**	**82.0**
25	53.9	90.3	5.55	0.51	10.88	73.3	79.7
30	46.2	93.0	6.63	0.58	11.43	76.6	77.7
35	39.4	94.7	7.42	0.64	11.59	78.7	75.9
40	33.7	96.2	8.86	0.69	12.84	81.5	74.5
45	27.8	97.2	9.85	0.74	13.31	83.1	73.1
50	22.8	97.9	10.79	0.79	13.65	84.4	71.9
55	18.8	98.5	12.84	0.82	15.65	86.4	71.0
60	15.1	99.0	12.85	0.86	14.94	88.1	70.0
**Male**							
05	99.04	12.67	1.13	0.07	16.14	54.2	91.7
10	96.3	30.7	1.38	0.13	10.61	62.0	86.4
15	90.9	45.7	1.68	0.20	8.4	66.4	81.0
20	85.3	57.5	2.01	0.26	7.73	70.3	76.9
**25**	**78.5**	**67.0**	**2.38**	**0.32**	**7.43**	**73.7**	**72.5**
30	71.1	74.5	2.78	0.39	7.12	76.6	68.6
**35**	**63.0**	**79.8**	**3.12**	**0.46**	**6.78**	**78.6**	**64.6**
40	56.1	84.1	3.53	0.52	6.78	80.6	61.9
45	49.2	84.8	4.03	0.58	6.94	82.6	59.5
50	42.5	90.7	4.55	0.63	7.22	84.3	57.2
55	36.6	92.9	5.08	0.69	7.36	85.7	55.2
60	30.5	94.8	5.83	0.73	7.98	87.3	53.6

Abbreviations: LR+: positive likelihood ratio; LR−: negative likelihood ratio; PPV: positive predictive value; NPV: negative predictive value; DOR: diagnostic odd ratios

## Discussion

With the growing epidemic of obesity, the prevalence rates of fatty liver have increased in both Eastern and Western countries [7,8]. Previous studies conducted in Western countries demonstrated that the risk factors of fatty liver disease include age, gender, and metabolic factors, such as central obesity, higher BMI, elevated fasting blood glucose, insulin, TG, and cholesterol, and lower HDL levels [9,22]. Our study also validates that Asian subjects are at risk of ultrasonogrphic fatty liver disease if they possess these metabolic aberrances.

Although Asians have a significantly lower BMI compared to other ethnic populations, they have a surprisingly high prevalence rate of fatty liver disease [23]. Moreover, Asian people have a significantly higher incidence of metabolic syndrome than other ethnic groups with similar BMI [24]. This may due to the more central adiposity and visceral fat deposition in Asian subjects [23]. In our study, fatty liver was diagnosed by ultrasonography in 44.5% of patients who received physical check-up in a single medical center from Taiwan. Notably, the prevalence rates of ultrasonogrphic fatty liver were 5.2% and 17.9% for subjects with BMIs of <17.5 kg/m^2^ and 17.5–22.4 kg/m^2^, respectively. This suggests that fatty liver disease is not uncommon in non-obese Asian subjects [23]. They may progress to steatohepatitis, cirrhosis, and even HCC if not diagnosed promptly. Moreover, previous studies have demonstrated that the development of HCC in patients with hepatic steatosis can occur in the absence of liver cirrhosis [25]. Consequently, the development of a feasible non-invasive screening marker is needed to identify high-risk groups of patients with fatty liver in non-obese populations, who are easily overlooked if there is a lack of clinical suspicion.

Fatty liver is not only a hepatic manifestation of metabolic syndrome, but it could also promote the development of metabolic-related extra-hepatic complications like cardiovascular disease (CVD), type 2 diabetes, chronic kidney disease, hypothyroidism, polycystic ovarian syndrome, osteoporosis, and colorectal cancer [1,9,26]. Notably, a growing body of evidence shows that patients with fatty liver disease have a significantly higher risk of CVD compared to general populations of the same age and gender [27,28]. Although CVD and fatty liver disease share similar risk factors, such as insulin resistance and central adiposity, fatty liver disease is independently associated with the presence of CVD [26]. This may be due to the increased inflammatory cytokines, insulin resistance, and free fatty acid promoted by the expanded and inflamed visceral fat mass in patients with fatty liver [27]. It highlights the critical role of fatty liver in determining the prognoses of subjects with metabolic syndrome. Hence, there is an urgent need to identify well-validated, quantitative, cost-effective, and non-invasive methods for the evaluation of fatty liver disease in clinical practice as well as epidemiological and clinical research.

FLI is a feasible marker that involves four clinical available parameters, and it is easily calculated in an office setting. It has been proven to correlate well with fatty liver diagnosed by ultrasonography [16]. Moreover, one study conducted with a large middle-aged non-diabetes European population demonstrated that FLI was correlated with insulin resistance, coronary heart disease, and early atherosclerosis [29]. Calori et al. further showed that FLI was associated with all-cause, hepatic-related, cardiovascular disease-related, and cancer mortality [30]. This suggests that FLI could be applied not only for screening fatty liver disease, but also for identifying high-risk groups of subjects for metabolic and cardiovascular disorders, which are critical public health issues that are worthy of concern. However, due to variations in ethnicity, dietary, and environmental factors, the cut-off for waist and BMI is different for Asian people [31]. Thus, FLI needs to be validated when used in a different population. Our study confirmed the significance of FLI as an indicator of ultrasonogrphic fatty liver and its close link to metabolic syndrome. It had better discriminative ability for identifying ultrasonogrphic fatty liver than other serum markers and could therefore be recommended for Asian subjects.

In our cohort, male subjects had a higher rate of ultrasonogrphic fatty liver compared to the female population in most subgroup analyses. There are several mechanisms that might explain this phenomenon. First, male subjects had higher BMI and showed more metabolic derangement, which are both important determinants of fatty liver disease. Second, in comparison to females, male subjects had more accelerating visceral adipose tissue expansion with the increase of age, which in turn facilitates the development of insulin resistance and fatty liver by the production of free fatty acid and adipocytokines [32]. Third, it is speculated that estrogen could suppress visceral adipose tissue and TG accumulation [33]. One recent study showed that estrogen receptor ligands reduced hepatic TG levels through the inhibition of liver X receptor transcriptional activity in a mouse model [34]. For the female population, the risk of obesity and metabolic syndrome markedly increase after menopause, implying a protective role of estrogen in the development of fatty liver disease [35]. This may explain the differences in the prevalence rate of ultrasonogrphic fatty liver in both genders decreasing after post-menopausal age in our cohort **(Fig. 1A).** Moreover, females had a similar prevalence rate of ultrasonogrphic fatty liver compared to males in subjects who were older than 70 years.

Compared to the cut-off values proposed by Bedogni et al., our findings were lower for both genders [16]. According to the metabolic syndrome criteria from the joint interim statement of the International Diabetes Federation Task Force on Epidemiology and Prevention, the cut-off values of WC, which is a major component of FLI, are set lower for Asian female subjects (80 cm) and for males (90 cm), respectively [31,32]. This might explain the lower cut-off values of FLI in our cohort, especially in female subjects. Another possible mechanism is the more central adiposity and higher incidence of fatty liver in non-obese Asians compared to the Western populations [24]. Nevertheless, further prospective studies are still needed to validate this result.

This study has a number of limitations that are worth noting. First, the study population had a higher socio-economic status, and subjects could afford the expense of a physical check-up, so the prevalence rate of ultrasonogrphic fatty liver may be higher than that of the general population. Second, some liver-related diseases, such as alcohol consumption, medication use, autoimmune liver disease, congenital liver disease, diabetes mellitus and hyperlipidemia were not documented in the study. Nevertheless, previous studies showed that these factors may only have a small effect on the result [36,37]. For example, Chen et al. demonstrated that the etiology of elevated serum ALT level due to alcohol consumption is as low as 0.8% in a Taiwanese community study [38]. Besides, from a National Health Interview Survey in Taiwan, there were 1.82% of the young adults and 4.82% of the middle-aged adults in Taiwan drank alcohol on a daily basis with the corresponding estimates for probable alcoholism were 2.40% and 2.27%, respectively [39]. This suggests that alcohol consumption only plays a very minor role in the development of fatty liver in Taiwan. Third, we adopted ultrasonography as a diagnostic tool for fatty liver. Its sensitivity decreased with while hepatic steatosis is less than 20%. It might result in underestimation of the rate of fatty liver disease. Although ultrasonography was performed by five 10-year-experience senior doctors with the same criteria, the inter-observation and intra-observation variations were unavailable due to the retrospective study design. Nevertheless, the strengths of this study are the large sample size and the detailed biochemistry data which provide robust data for the external validation of FLI in predicting ultrasonogrphic fatty liver in Asian subjects.

In conclusion, FLI could accurately identify ultrasonogrphic fatty liver in a large-scale population in Taiwan but the optimal cut-off values are lower than the Western people. Besides, the cut-off value was also noted lower in females. For the males, the optimal cut-off values are the FLI <25 to rule out and FLI ≥35 to rule in ultrasonogrphic fatty liver. For the females, FLI <10 for exclusion and FLI ≥ 20 for inclusion of ultrasonogrphic fatty liver are chosen.

## Supporting Information

S1 Table Comparison of demographic characteristics between male and female subjects. (DOCX)

S2 Table Comparison of prevalence rates of ultrasonogrphic fatty liver between male and female subjects stratified by body mass index. (DOCX)

S3 Table Factors associated with ultrasonogrphic fatty liver in different populations by multivariate analysis in model II. (DOCX)

## References

[1] Armstrong MJ, Adams LA, Canbay A, Syn WK. Extrahepatic complications of nonalcoholic fatty liver disease. Hepatology 2014;59: 1174–1197. 10.1002/hep.26717 24002776

[2] Vernon G, Baranova A, Younossi ZM. Systematic review: the epidemiology and natural history of non-alcoholic fatty liver disease and non-alcoholic steatohepatitis in adults. Aliment Pharmacol Ther 2011;34: 274–285. 10.1111/j.1365-2036.2011.04724.x 21623852

[3] Nascimbeni F, Pais R, Bellentani S, Day CP, Ratziu V, Loria P, et al From NAFLD in clinical practice to answers from guidelines. J Hepatol 2013;59: 859–871. 10.1016/j.jhep.2013.05.044 23751754

[4] Loomba R, Abraham M, Unalp A, Wilson L, Lavine J, Doo E, et al Association between diabetes, family history of diabetes, and risk of nonalcoholic steatohepatitis and fibrosis. Hepatology 2012;56: 943–951. 10.1002/hep.25772 22505194 PMC3407289

[5] Lee JY, Kim KM, Lee SG, Yu E, Lim YS, Lee HC, et al Prevalence and risk factors of non-alcoholic fatty liver disease in potential living liver donors in Korea: a review of 589 consecutive liver biopsies in a single center. J Hepatol 2007;47: 239–244. 17400323 10.1016/j.jhep.2007.02.007

[6] Fan JG, Farrell GC. Epidemiology of non-alcoholic fatty liver disease in China. J Hepatol 2009;50: 204–210. 10.1016/j.jhep.2008.10.010 19014878

[7] Wong VW. Nonalcoholic fatty liver disease in Asia: a story of growth. J Gastroenterol Hepatol 2013;28: 18–23. 10.1111/jgh.12259 23094755

[8] Younossi ZM, Stepanova M, Afendy M, Fang Y, Younossi Y, Mir H, et al Changes in the prevalence of the most common causes of chronic liver diseases in the United States from 1988 to 2008. Clin Gastroenterol Hepatol 2011;9: 524–530. 10.1016/j.cgh.2011.03.020 21440669

[9] Chalasani N, Younossi Z, Lavine JE, Diehl AM, Brunt EM, Cusi K, et al The diagnosis and management of non-alcoholic fatty liver disease: practice guideline by the American Gastroenterological Association, American Association for the Study of Liver Diseases, and American College of Gastroenterology. Gastroenterology 2012;142: 1592–1609. 10.1053/j.gastro.2012.04.001 22656328

[10] Angulo P, Bugianesi E, Bjornsson ES, Charatcharoenwitthaya P, Mills PR, Barrera F, et al Simple noninvasive systems predict long-term outcomes of patients with nonalcoholic fatty liver disease. Gastroenterology 2013;145: 782–789. 10.1053/j.gastro.2013.06.057 23860502 PMC3931256

[11] Charlton MR, Burns JM, Pedersen RA, Watt KD, Heimbach JK, Dierkhising RA. Frequency and outcomes of liver transplantation for nonalcoholic steatohepatitis in the United States. Gastroenterology 2011;141: 1249–1253. 10.1053/j.gastro.2011.06.061 21726509

[12] Welzel TM, Graubard BI, Quraishi S, Zeuzem S, Davila JA, El-Serag HB, et al Population-attributable fractions of risk factors for hepatocellular carcinoma in the United States. Am J Gastroenterol 2013;108: 1314–1321. 10.1038/ajg.2013.160 23752878 PMC4105976

[13] Hyysalo J, Mannisto VT, Zhou Y, Arola J, Karja V, Leivonen M, et al A population-based study on the prevalence of NASH using scores validated against liver histology. J Hepatol 2014;60: 839–846. 10.1016/j.jhep.2013.12.009 24333862

[14] Pais R, Charlotte F, Fedchuk L, Bedossa P, Lebray P, Poynard T, et al A systematic review of follow-up biopsies reveals disease progression in patients with non-alcoholic fatty liver. J Hepatol 2013;59: 550–556. 10.1016/j.jhep.2013.04.027 23665288

[15] Wong VW, Chu WC, Wong GL, Chan RS, Chim AM, Ong A, et al Prevalence of non-alcoholic fatty liver disease and advanced fibrosis in Hong Kong Chinese: a population study using proton-magnetic resonance spectroscopy and transient elastography. Gut 2012;61: 409–415. 10.1136/gutjnl-2011-300342 21846782

[16] Bedogni G, Bellentani S, Miglioli L, Masutti F, Passalacqua M, Castiglione A, et al The Fatty Liver Index: a simple and accurate predictor of hepatic steatosis in the general population. BMC Gastroenterol 2006;6: 33 17081293 10.1186/1471-230X-6-33 PMC1636651

[17] Koehler EM, Schouten JN, Hansen BE, Hofman A, Stricker BH, Janssen HL, et al External validation of the fatty liver index for identifying nonalcoholic fatty liver disease in a population-based study. Clin Gastroenterol Hepatol 2013;11: 1201–1204. 10.1016/j.cgh.2012.12.031 23353640

[18] Bedogni G, Kahn HS, Bellentani S, Tiribelli C. A simple index of lipid overaccumulation is a good marker of liver steatosis. BMC Gastroenterol 2010;10: 98 10.1186/1471-230X-10-98 20738844 PMC2940930

[19] Sanyal AJ. AGA technical review on nonalcoholic fatty liver disease. Gastroenterology 2002;123: 1705–1725. 12404245 10.1053/gast.2002.36572

[20] Hernaez R, Lazo M, Bonekamp S, Kamel I, Brancati FL, Guallar E, et al Diagnostic accuracy and reliability of ultrasonography for the detection of fatty liver: a meta-analysis. Hepatology 2011;54: 1082–1090. 10.1002/hep.24452 21618575 PMC4197002

[21] Dasarathy S, Dasarathy J, Khiyami A, Joseph R, Lopez R, McCullough AJ. Validity of real time ultrasound in the diagnosis of hepatic steatosis: a prospective study. J Hepatol 2009;51: 1061–1067. 10.1016/j.jhep.2009.09.001 19846234 PMC6136148

[22] Wu WC, Wu CY, Wang YJ, Hung HH, Yang HI, Kao WY, et al Updated thresholds for serum alanine aminotransferase level in a large-scale population study composed of 34 346 subjects. Aliment Pharmacol Ther 2012;36: 560–568. 10.1111/j.1365-2036.2012.05224.x 22817613

[23] Koehler EM, Schouten JN, Hansen BE, van Rooij FJ, Hofman A, Stricker BH, et al Prevalence and risk factors of non-alcoholic fatty liver disease in the elderly: results from the Rotterdam study. J Hepatol 2012;57: 1305–1311. 10.1016/j.jhep.2012.07.028 22871499

[24] Wong RJ, Ahmed A. Obesity and non-alcoholic fatty liver disease: Disparate associations among Asian populations. World J Hepatol 2014;6: 263–273. 10.4254/wjh.v6.i5.263 24868320 PMC4033284

[25] Palaniappan LP, Wong EC, Shin JJ, Fortmann SP, Lauderdale DS. Asian Americans have greater prevalence of metabolic syndrome despite lower body mass index. Int J Obes (Lond) 2011;35: 393–400. 10.1038/ijo.2010.152 20680014 PMC2989340

[26] Treeprasertsuk S, Leverage S, Adams LA, Lindor KD, St Sauver J, Angulo P. The Framingham risk score and heart disease in nonalcoholic fatty liver disease. Liver Int 2012;32: 945–950. 10.1111/j.1478-3231.2011.02753.x 22299674 PMC3348257

[27] Paradis V, Zalinski S, Chelbi E, Guedj N, Degos F, Vilgrain V, et al Hepatocellular carcinomas in patients with metabolic syndrome often develop without significant liver fibrosis: a pathological analysis. Hepatology 2009;49: 851–859. 10.1002/hep.22734 19115377

[28] Targher G, Day CP, Bonora E. Risk of cardiovascular disease in patients with nonalcoholic fatty liver disease. N Engl J Med 2010;363: 1341–1350. 10.1056/NEJMra0912063 20879883

[29] Kim D, Choi SY, Park EH, Lee W, Kang JH, et al Nonalcoholic fatty liver disease is associated with coronary artery calcification. Hepatology 2012;56: 605–613. 10.1002/hep.25593 22271511 PMC3830979

[30] Gastaldelli A, Kozakova M, Hojlund K, Flyvbjerg A, Favuzzi A, Kim W, et al Fatty liver is associated with insulin resistance, risk of coronary heart disease, and early atherosclerosis in a large European population. Hepatology 2009;49: 1537–1544. 10.1002/hep.22845 19291789

[31] Calori G, Lattuada G, Ragogna F, Garancini MP, Crosignani P, Villa M, et al Fatty liver index and mortality: the Cremona study in the 15th year of follow-up. Hepatology 2011;54: 145–152. 10.1002/hep.24356 21488080

[32] Alberti KG, Eckel RH, Grundy SM, Zimmet PZ, Cleeman JI, Donato KA, et al Harmonizing the metabolic syndrome: a joint interim statement of the International Diabetes Federation Task Force on Epidemiology and Prevention; National Heart, Lung, and Blood Institute; American Heart Association; World Heart Federation; International Atherosclerosis Society; and International Association for the Study of Obesity. Circulation 2009;120: 1640–1645. 10.1161/CIRCULATIONAHA.109.192644 19805654

[33] Ibrahim MM. Subcutaneous and visceral adipose tissue: structural and functional differences. Obes Rev 2010;11: 11–18. 10.1111/j.1467-789X.2009.00623.x 19656312

[34] Ayonrinde OT, Olynyk JK, Beilin LJ, Mori TA, Pennell CE, de Klerk N, et al Gender-specific differences in adipose distribution and adipocytokines influence adolescent nonalcoholic fatty liver disease. Hepatology 2011;53: 800–809. 10.1002/hep.24097 21374659

[35] Han SI, Komatsu Y, Murayama A, Steffensen KR, Nakagawa Y, Nakajima Y, et al Estrogen receptor ligands ameliorate fatty liver through a nonclassical estrogen receptor/Liver X receptor pathway in mice. Hepatology 2014;59: 1791–1802. 10.1002/hep.26951 24277692

[36] Yang JD, Abdelmalek MF, Pang H, Guy CD, Smith AD, Diehl AM, et al Gender and menopause impact severity of fibrosis among patients with nonalcoholic steatohepatitis. Hepatology 2014;59: 1406–1414. 10.1002/hep.26761 24123276 PMC3966932

[37] Cheng YL, Wang YJ, Kao WY, Chen PH, Huo TI, Huang YH, et al Inverse association between hepatitis B virus infection and fatty liver disease: a large-scale study in populations seeking for check-up. PLoS One 2013;8: e72049 10.1371/journal.pone.0072049 23991037 PMC3750031

[38] Chen CH, Huang MH, Yang JC, Nien CK, Yang CC, Yeh YH, et al Prevalence and etiology of elevated serum alanine aminotransferase level in an adult population in Taiwan. J Gastroenterol Hepatol 2007;22: 1482–1489. 17716352 10.1111/j.1440-1746.2006.04615.x

[39] Lin CY, Chen KH, Chang HY, Tseng FY, Chen CY. The relationship between the pattern of alcohol consumption and healthcare utilization in Taiwan. Taiwan J Public Health 2014;33: 197–208.

