# Supplementary material for: External Validation of Fatty Liver Index for Identifying Ultrasonographic Fatty Liver in a Large-Scale Cross-Sectional Study in Taiwan
Source: PLoS One. 2015 Mar 17;10(3):e0120443. doi: 10.1371/journal.pone.0120443 (PMC4363626; doi:10.1371/journal.pone.0120443)
Supplement: S1 Table — (DOCX) [file pone.0120443.s001.docx]

**Table S1. Comparison of demographic characteristics between male and female subjects**

|  | **Male**  **(n=16098)** | **Female**  **(n=13699)** | **P** |
| --- | --- | --- | --- |
| **Age, years** | 53.0±13.7 | 51.3±12.6 | <0.001 |
| **BMI, kg/m^2^** | 24.64±3.38 | 22.85±3.57 | <0.001 |
| **WC, cm** | 87.5±9.2 | 79.5±9.8 | <0.001 |
| **SBP, mmHg** | 126.8±17.1 | 121.3±19.8 | <0.001 |
| **DBP, mmHg** | 79.6±14.4 | 75.2±13.7 | <0.001 |
| **Fasting Glucose, mg/dL** | 97.6±26.8 | 93.0±21.9 | <0.001 |
| **Cholesterol, mg/dL** | 198.1±36.3 | 200.5±37.8 | <0.001 |
| **LDL, mg/dL** | 127.0±32.2 | 123.4±33.6 | <0.001 |
| **HDL, mg/dL** | 47.9±12.1 | 60.5±15.2 | <0.001 |
| **TG, mg/dL** | 150.1±98.6 | 107.3±66.8 | <0.001 |
| **ALT, U/L** | 30.9±24.2 | 22.5±18.5 | <0.001 |
| **AST, U/L** | 24.1±13.3 | 21.9±13.0 | <0.001 |
| **GGT, IU/L** | 29.8±43.2 | 18.8±26.3 | <0.001 |
| **Platelet, 1000/mm^3^** | 241.11±57.50 | 260.06±61.95 | <0.001 |
| **Fatty liver, yes/no (%)** | 8710/7388 (54.1/45.9) | 4545/9154 (33.2/66.8) | <0.001 |
| **FLI** | 35.35±24.68 | 17.70±19.70 | <0.001 |

Continuous variables are expressed as mean ± standard deviation

Abbreviations: BMI, body mass index; WC, waist circumference; SBP, systolic blood pressure; DBP, diastolic blood pressure; LDL, low-density lipoprotein; HDL, high-density lipoprotein; TG, triglyceride; ALT, alanine aminotransferase; AST, aspartate aminotransferase; GGT, gamma-glutamyl transferase; FLI, fatty liver index
