# Supplementary material for: External Validation of Fatty Liver Index for Identifying Ultrasonographic Fatty Liver in a Large-Scale Cross-Sectional Study in Taiwan
Source: PLoS One. 2015 Mar 17;10(3):e0120443. doi: 10.1371/journal.pone.0120443 (PMC4363626; doi:10.1371/journal.pone.0120443)
Supplement: S2 Table — (DOCX) [file pone.0120443.s002.docx]

**Table S2. Comparison of prevalence rates of ultrasonogrphic fatty liver between male and female subjects stratified by body mass index**

| **Body mass index** | **All subjects, fatty liver**  **yes/no (%)** | **Male,**  **fatty liver**  **yes/no (%)** | **Female,**  **fatty liver**  **yes/no (%)** | **P** |
| --- | --- | --- | --- | --- |
| < 17.5 kg/m^2^ | 29/524 (5.2%/94.8%) | 12/139 (7.9%/92.1%) | 17/385 (4.2%/95.8%) | 0.089 |
| 17.5-22.4 kg/m^2^ | 1821/8361 (17.9%/82.1%) | 820/2823 (22.5%/77.5%) | 1001/5538 (15.3%/84.7%) | <0.001 |
| 22.5-24.9 kg/m^2^ | 4141/4923 (45.7%/54.3%) | 2750/2751 (50.0%/50.0%) | 1391/2172 (39.0%/61.0%) | <0.001 |
| 25.0-29.9 kg/m^2^ | 5995/2546 (70.2%/29.8%) | 4302/1582 (73.1%/26.9%) | 1693/964 (63.7%/36.3%) | <0.001 |
| ≧30 kg/m^2^ | 1269/188 (87.1%/12.9%) | 826/93 (89.9%/10.1%) | 443/95 (82.3%/17.7%) | <0.001 |
