# Supplementary material for: External Validation of Fatty Liver Index for Identifying Ultrasonographic Fatty Liver in a Large-Scale Cross-Sectional Study in Taiwan
Source: PLoS One. 2015 Mar 17;10(3):e0120443. doi: 10.1371/journal.pone.0120443 (PMC4363626; doi:10.1371/journal.pone.0120443)
Supplement: S3 Table — (DOCX) [file pone.0120443.s003.docx]

**Table S3. Factors associated with ultrasonogrphic fatty liver in different populations by multivariate analysis in model II**

|  | **Odds Ratio** | **95% Confidence level** | ***P* value** |
| --- | --- | --- | --- |
| **All subjects** |  |  |  |
| **BMI, kg/m^2^** | **1.223** | **1.205-1.242** | **<0.0001** |
| **WC, cm** | **1.034** | **1.029-1.040** | **<0.0001** |
| Fasting Glucose, mg/dL | 1.009 | 1.007-1.010 | <0.0001 |
| LDL, mg/dL | 1.006 | 1.006-1.007 | <0.0001 |
| HDL, mg/dL | 0.985 | 0.983-0.988 | <0.0001 |
| **TG, mg/dL** | **1.007** | **1.006-1.007** | **<0.0001** |
| **GGT, U/L** | **1.003** | **1.002-1.004** | **<0.0001** |
| **Females** |  |  |  |
| **BMI, kg/m^2^** | **1.224** | **1.198-1.250** | **<0.0001** |
| **WC, cm** | **1.021** | **1.013-1.028** | **<0.0001** |
| Fasting Glucose, mg/dL | 1.011 | 1.009-1.014 | <0.0001 |
| LDL, mg/dL | 1.006 | 1.005-1.008 | <0.0001 |
| HDL, mg/dL | 0.984 | 0.981-0.988 | <0.0001 |
| **TG, mg/dL** | **1.008** | **1.007-1.009** | **<0.0001** |
| **GGT, U/L** | **1.006** | **1.004-1.008** | **<0.0001** |
| **Males** |  |  |  |
| **BMI, kg/m^2^** | **1.212** | **1.185-1.240** | **<0.0001** |
| **WC, cm** | **1.046** | **1.038-1.055** | **<0.0001** |
| Fasting Glucose, mg/dL | 1.007 | 1.005-1.008 | <0.0001 |
| LDL, mg/dL | 1.006 | 1.005-1.007 | <0.0001 |
| HDL, mg/dL | 0.988 | 0.984-0.991 | <0.0001 |
| **TG, mg/dL** | **1.006** | **1.006-1.007** | **<0.0001** |
| **GGT, U/L** | **1.002** | **1.001-1.003** | **<0.0001** |

Abbreviations: BMI, body mass index; WC, waist circumference; LDL, low-density lipoprotein; HDL, high-density lipoprotein; TG, triglyceride; GGT, gamma-glutamyl transferase;
